# Joint Modeling of Immune Reconstitution Post Haploidentical Stem Cell Transplantation in Pediatric Patients With Acute Leukemia Comparing CD34^+^-Selected to CD3/CD19-Depleted Grafts in a Retrospective Multicenter Study

**DOI:** 10.3389/fimmu.2018.01841

**Published:** 2018-08-14

**Authors:** Emilia Salzmann-Manrique, Melanie Bremm, Sabine Huenecke, Milena Stech, Andreas Orth, Matthias Eyrich, Ansgar Schulz, Ruth Esser, Thomas Klingebiel, Peter Bader, Eva Herrmann, Ulrike Koehl

**Affiliations:** ^1^Department of Medicine, Institute of Biostatistics and Mathematical Modeling, Johann Wolfgang Goethe-University, Frankfurt, Germany; ^2^Pediatric Hematology and Oncology, Johann Wolfgang Goethe-University, Frankfurt, Germany; ^3^University of Applied Sciences Frankfurt, Frankfurt, Germany; ^4^Pediatric Hematology and Oncology, University of Wuerzburg, Wuerzburg, Germany; ^5^Pediatric Hematology and Oncology, University of Ulm, Ulm, Germany; ^6^Institute of Cellular Therapeutics Hannover Medical School, Hannover, Germany; ^7^Institute of Clinical Immunology, University of Leipzig, Leipzig, Germany; ^8^Fraunhofer Institute of Cellular Therapy and Immunology, Leipzig, Germany

**Keywords:** immune reconstitution, allogeneic stem cell transplantation, CD34 selection, CD3/19 depletion, children

## Abstract

Rapid immune reconstitution (IR) following stem cell transplantation (SCT) is essential for a favorable outcome. The optimization of graft composition should not only enable a sufficient IR but also improve graft vs. leukemia/tumor effects, overcome infectious complications and, finally, improve patient survival. Especially in haploidentical SCT, the optimization of graft composition is controversial. Therefore, we analyzed the influence of graft manipulation on IR in 40 patients with acute leukemia in remission. We examined the cell recovery post haploidentical SCT in patients receiving a CD34^+^-selected or CD3/CD19-depleted graft, considering the applied conditioning regimen. We used joint model analysis for overall survival (OS) and analyzed the dynamics of age-adjusted leukocytes; lymphocytes; monocytes; CD3^+^, CD3^+^CD4^+^, and CD3^+^CD8^+^ T cells; natural killer (NK) cells; and B cells over the course of time after SCT. Lymphocytes, NK cells, and B cells expanded more rapidly after SCT with CD34^+^-selected grafts (*P* = 0.036, *P* = 0.002, and *P* < 0.001, respectively). Contrarily, CD3^+^CD4^+^ helper T cells recovered delayer in the CD34 selected group (*P* = 0.026). Furthermore, reduced intensity conditioning facilitated faster immune recovery of lymphocytes and T cells and their subsets (*P* < 0.001). However, the immune recovery for NK cells and B cells was comparable for patients who received reduced-intensity or full preparative regimens. Dynamics of all cell types had a significant influence on OS, which did not differ between patients receiving CD34^+^-selected and those receiving CD3/CD19-depleted grafts. In conclusion, cell reconstitution dynamics showed complex diversity with regard to the graft manufacturing procedure and conditioning regimen.

## Introduction

The transplantation of allogeneic hematopoietic stem cells [stem cell transplantation (SCT)] provides a curative treatment option for patients suffering from high-risk acute leukemia and other hematological malignancies (1–4). The success of SCT is dependent on the rapid reconstitution of immune-competent cells, which is influenced by different factors (5–10). Adequate immune recovery may result in the eradication of residual malignant cells (11) and can protect the patient from infectious complications (12, 13). Allogeneic SCT is mostly performed from an HLA-matched related or unrelated donor. For patients who lack a suitable donor, a haploidentical SCT and umbilical cord blood SCT (14) serve as an alternative. Haploidentical SCT (haplo-SCT) carries an increased risk of both graft vs. host disease (GvHD) and graft rejection compared to HLA-matched SCT but shows a decreased relapse rate (2, 15–17). GvHD is mainly induced by donor T cells (18, 19). Therefore, T cells need to be depleted prior to the infusion of the haploidentical graft. Different strategies for the purification of haploidentical grafts are available, such as the positive selection of CD34^+^ stem cells leading to indirect T-cell depletion (2, 15, 17) and the direct depletion of CD3^+^ T cells or TCR alpha/beta T cells, both in combination with CD19^+^ B-cell removal (20–23). CD34^+^-selected grafts contain highly purified stem cells and can be grafted in the absence of GvHD prophylaxis (24). CD3/CD19 or TCR alpha/beta-depleted grafts contain hematopoietic progenitors, natural killer (NK) cells, and antigen-presenting cells, probably leading to an enhanced graft vs. leukemia effect (16, 20, 23).

To date, studies about cellular immune reconstitution (IR) in pediatric patients post haplo-SCT are mainly done as single center studies and have long-term monitoring intervals, leading to small and heterogeneous patient cohorts. Therefore, in a retrospective multicenter study (Frankfurt, Ulm, Wuerzburg), we examined the IR post haplo-SCT in a relatively homogeneous group of 40 pediatric patients suffering from acute leukemia. We analyzed and compared the regeneration of various leukocyte subpopulations from children receiving CD34^+^-selected and CD3/CD19-depleted grafts. Our study considered age-adjusted leukocytes subpopulations as well as the individual progress of IR of each patient. In this respect, we were able to set up a joint model for IR, which allowed modeling of the continuous recovery process over time post SCT.

## Patients and Methods

### Patients

In this retrospective study, we included 40 pediatric patients suffering from high-risk leukemia transplanted in complete remission (CR) with HLA-haploidentical peripheral blood (PB) stem cells between 1997 and 2012. Informed consent was obtained from all patients/parents, and the retrospective study was approved by the local ethic committee (Frankfurt, D Ethical No: 499/16). Inclusion criteria for patient recruitment in the three centers were as follows: (1) patients with acute lymphoblastic (ALL) or acute myeloblastic leukemia (AML), (2) a PBSC graft from a haploidentical donor processed with either positive CD34^+^ immunomagnetic selection (PBSC_CD34sel_) or CD3/CD19 depletion (PBSC_CD3/CD19dep_) and (3) in case of multiple transplantation, inclusion criteria requires a >6-month interval between the last and penultimate transplant. Only immune monitoring after the last transplantation was considered in these cases.

Depending on the underlying disease, either myeloablative conditioning regimens (MAC) based on total body irradiation (12 Gy TBI) or busulphan, or a reduced-intensity conditioning regimen (RIC) with fludarabine, thiotepa, and melphalan were used. The positive selection of CD34^+^ PB stem cells with immunomagnetic microbeads and the direct depletion of T and B cells with-coated microbeads under good manufacturing practice (GMP) were performed with-coated microbeads using a CliniMACS (Miltenyi Biotec, Bergisch-Gladbach, Germany) device under GMP as we described previously (23, 25). For prophylaxis of GvHD, OKT3 or anti-thymocyte globulin was given. Posttransplantation, the GvHD prophylaxis consisted of mycophenolate mofetil in cases of patients receiving PBSC_CD3/CD19dep_ grafts, while for patients transplanted with PBSC_CD34sel_ stem cells, no GvHD prophylaxis was given. The grade of acute GvHD was defined according to published consensus criteria (26). Engraftment of neutrophils was defined as the first of 3 days with a neutrophil count >500 cells/μl.

### Flow Cytometric Analyses for Monitoring of IR

In all three centers, PB samples were collected regularly post haplo-SCT (>10 times/year) using EDTA tubes and analyzed the same day to monitor IR. The absolute numbers of leukocytes, CD14^+^ monocytes, lymphocytes, CD3^+^ T cells, CD3^+^CD4^+^ helper T cells, CD3^+^CD8^+^ cytotoxic T cells, CD3^−^CD56^+^ NK cells and CD19^+^ B cells were determined by flow cytometry in a lyse-no-wash procedure as we described previously (8, 9, 25, 27). Briefly, two tubes with 100 µl of PB were labeled with tetraCHROME (Frankfurt) or manually pipetted (Wuerzburg, Ulm) with combinational monoclonal antibody reagents for CD45/CD4/CD8/CD3 and CD45/CD56/CD19/CD3 conjugated with fluorescein isothiocyanate, phycoerythrin (PE), phycoerythrin Texas red (ECD), phycoerythrin-cyanine 5.1 (PC5), PerCp, and APC, respectively. The accredited tetraCHROME antibodies included anti-CD45 (clone: B3821F4A), anti-CD4 (clone: SFCI12T4D11), anti-CD8 (clone: SFCI21Thy2D3), anti-CD3 (clone: UCHT1), anti-CD56 (clone: N901) and anti-CD19 (clone: J3-119). Monocytes (clone: RMO52) were detected by adding phycoerythrin-cyanine 7 (PC7) labeled anti-CD14 monoclonal antibodies to the first tube or by a separate CD45/CD14 analysis. All reagents are purchased from Beckman Coulter^®^ Immunotech (Marseilles, France) or BD/Pharmigen (Heidelberg). The samples were measured with an FC500™ (Coulter) or a FACSCalibur/Canto-II (BD) flow cytometer. Absolute cell counts were calculated from the percentage values using a dual-platform approach.

For assessment of quality control, flow-set™ fluorospheres or similar products were used to set up the photomultiplier tube values weekly. Fluorescence overlap was compensated for, and both the flow cytometer optical alignment and the fluidic stability were tested daily as we described previously. Control cells (i.e., Immuno-Trol™) were used for verification (27). During the study, the values of all controls remained within the designated limits.

### Statistical Analysis

Sequential measurements of eight leukocyte subpopulations after haplo-SCT were used to compare the dynamics of IR between patient cohorts receiving PBSC_CD34sel_ or PBSC_CD3/CD19dep_ grafts in relation to outcome. We characterized the IR dynamics as a continuous process over time and took the strong age-dependency of IR into account (27–35) by normalizing each quantification value with the corresponding age-specific expected mean value (see Table S1 in Supplementary Material). Furthermore, the regression models used normalized levels of the leukocyte subpopulations after log-transformation to obtain an approximately Gaussian distribution for the residuals.

Then, each of the eight leukocyte subpopulations and patient survival were analyzed with a joint model for longitudinal and time-to-event data (36). Thereby, the whole recovery process over time post SCT was assessed. As immune recovery after SCT is not linear in time, the longitudinal submodel was a linear mixed-effect model using B-splines of the third order with two inner knots (37). The time-to-event outcome was overall survival (OS), which was assessed with a Cox proportional hazard submodel (38). Furthermore, the Kaplan-Meier method, standard Cox proportional hazard regression and the log-rank test were used for analyzing OS.

In addition, patient characteristics are described by median and range. Fisher’s exact test, the Wilcoxon-Mann-Whitney test and the Kruskal-Wallis test were used to compare characteristics between the two groups.

All tests were two sided, and *P* < 0.05 was considered significant. Statistics analysis used the R-3.2.5 (R Foundation for Statistical Computing, Vienna, Austria) with the JM package for model fitting (39) and ‘BIAS for Windows’ v11.02 (Epsilon-Verlag, Frankfurt, Germany).

## Results

**Figure 1:**
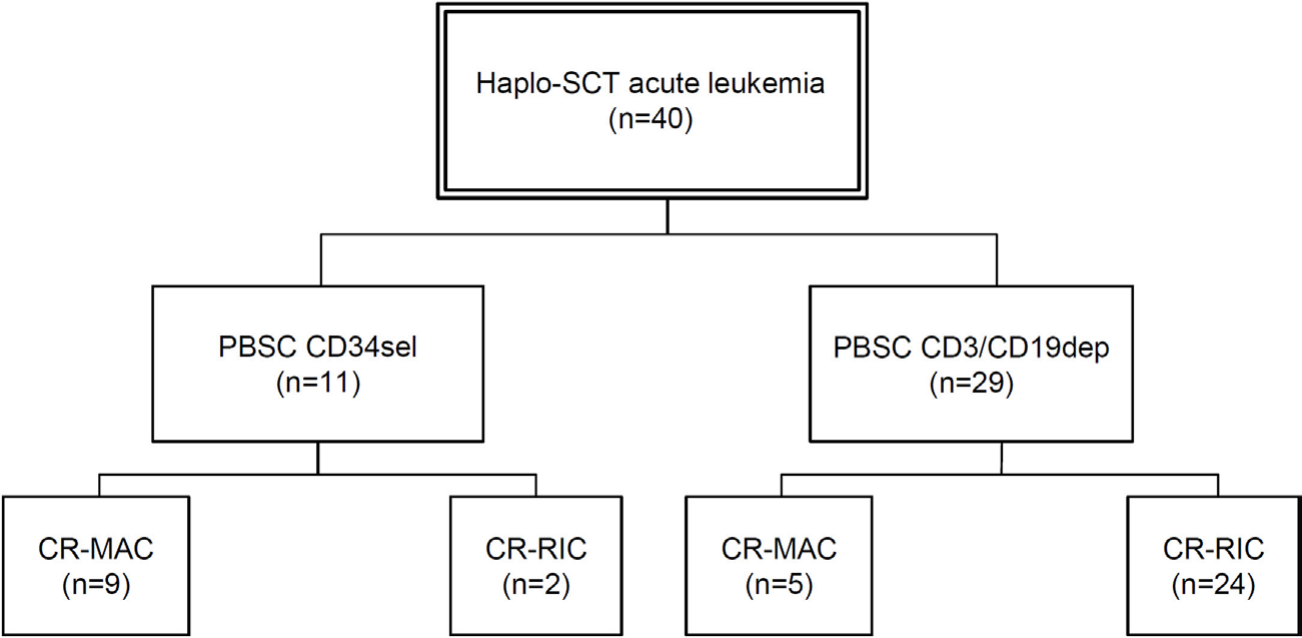
Flow chart with patient classification. Abbreviations: Haplo-SCT, haploidentical stem cell transplantation; PBSC, peripheral blood stem cells; CD34sel, CD34^+^ selection; CD3/CD19dep, CD3/CD19^+^ depletion; CR, complete remission; MAC, myeloablative conditioning; RIC, reduced-intensity conditioning.

The study cohort included 40 patients, with 11 patients receiving PBSC_CD34sel_ and 29 patients receiving PBSC_CD3/CD19dep_ in CR at transplantation time. Table 1 and Figure 1 give detailed information about both the patients and the transplant characteristics.

**Table 1:** Patient’s characteristics.

	CD34 sel	CD3/CD19 dep	*P*
		
	*n* = 11	*n* = 29	
SCT time period	August 1997–January 2005	April 2005–March 2012	
IR follow-up, median (range) (months)	5.7 (2.3–160.3)	8 (1.4–138)	
Survival follow-up, median (range) (months)	149 (72–160.3)	101 (44–138)	

**Patient-related factors**			
Age at SCT, median (range) years	7.5 (3.5–23)	10.6 (1.3–26)	0.437
Sex (male/female)	8/3	20/9	1
BMI at SCT, median (range) kg/m^2^	17.9 (14.3–20.2)	17.3 (11.9–28.7)	0.802

**Disease-related factors**			
Diagnosis			0.158
AML	2	13	
M0/M1/M2/M4/M5/M6/M7	0/0/1/1/0/0/0	2/2/3/1/2/1	
No data		2	
ALL	9	16	
T-ALL/BCP-ALL/Bipheno-ALL	3/6/0	7/7/2	
Status at SCT			
CR1/CR2/ ≥ CR3	2/5/4	8/12/9	0.820
Time from diagnosis to SCT, median (range) years	2.4 (0.7–4.2)	1.3 (0.3–5.7)	0.417

**Donor-related factors**			
Age, median (range) years	38 (28–42)	37 (24–51)	0.628
Sex (male/female)	9/2	16/13	0.158
Patient–donor sex			0.547
Female–female	1	5	
Male–male	7	12	
Male–female	1	8	
Female–male	2	4	
ABO compatibility			
Compatible	1	20	
Incompatible	3	7	
No data	7	2	
CMV status (recipient–donor)			
Positive–positive	2	11	
Negative–positive	–	9	
Positive–negative	–	1	
Negative–Negative	2	4	
No data	7	4	

**Transplanted-related factors**			
SCT-number			0.732
First/second/third	2/5/4	8/12/9	
Conditioning regimen			<0.001^a^
Standard myeloablative	9	5	
TBI-based	6	4	
Chemo-based	3	1	
Reduced myeloablative	2	24	
Flud/TT/Mel	2	24	
Serotherapy			0.005
Without	–	2	
ATG	8	5	
OKT3	3	21	
Graft vs. host disease prophylaxis			<0.001
Without	11	3	
MMF	–	25	
CSA, MTX	–	1	
Graft’s composition			
CD34^+^, median (range) × 10^6^/kg BW	15.3 (4.9–36.9)	10.1 (4.8–20.7)	0.009
CD3^+^, median (range) × 10^3^/kg BW	1.0 (0.4–3)	8.9 (0–5,000)	<0.001

**Treatment post-SCT**			
DLI	3	12	0.415
Number of DLI infusions 1/2/3/4/5	1/0/0/0/2	5/1/3/2/1	

*SCT, stem cell transplantation; IR, immune reconstitution; BMI, body mass index; ALL, acute lymphoblastic leukemia; AML, acute myeloid leukemia; M0, AML minimally differentiated; M1, AML without maturation; M2, AML with granulocytic maturation; M4, acute myelomonocytic leukemia; M5, acute monoblastic leukemia or acute monocytic leukemia; M6, acute erythroid leukemia; M7, acute megakaryoblastic leukemia; T-ALL, T cell ALL; BCP-ALL, B-cell precursor; B pheno ALL, biphenotypic/bilinear ALL; CR1, first complete remission; CR2, second complete remission; CR3, third complete remission; CMV, cytomegalovirus; TBI, total body irradiation; Flud, fludarabine; TT, thiotepa; Mel, melphalan; ATG, antithymocyte globuline; OKT3, anti-CD3 antibody; MMF, mycophenolate mofetil; MTX, methotrexate; CSA, cycloyporin A; BW, body weight*.

*^a^P-value for comparison of standard vs. reduced myeloablative conditioning regimen*.

### Dynamics of Immune Recovery

Our joint modeling approach primarily compared two graft manipulations (PBSC_CD34sel_ vs. PBSC_CD3/CD19dep_) considering the conditioning regimen (MAC vs. RIC).

**Figure 2:**
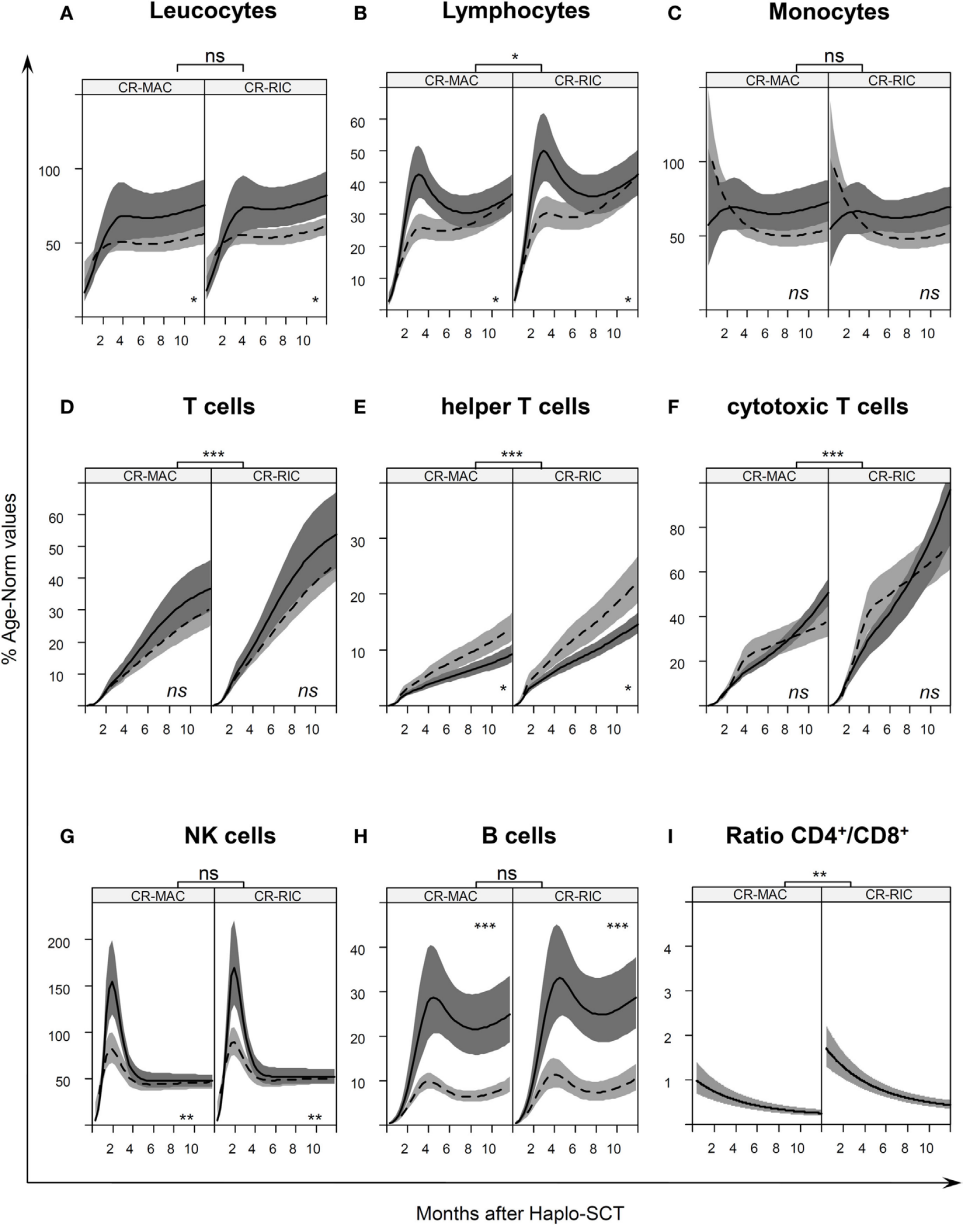
Age-normalized immune reconstitution (IR) in the first year after haplo-stem cell transplantation (SCT) IR normalized by age-specific norm values (27) Table S1 in Supplementary Material, of **(A)** leukocytes, **(B)** lymphocytes, **(C)** monocytes, **(D)** T cells, **(E)** helper T cells, **(F)** cytotoxic T cells, **(G)** natural killer, and **(H)** B cells in the first year after transplantation. The curves illustrate the splines of the longitudinal trajectory of IR after SCT based on our joint models with point wise 95% confidence regions. The CR-MAC and CR-RIC panels (left and right panel, respectively) exhibit the predicted IR for patients in any remission prior to SCT according to the type of graft PBSC_CD34sel_ (solid line with dark gray confidence regions) or PBSC_CD3/CD19dep_ (dashed line with light gray confidence regions). **(I)** CD4/CD8 ratio of the absolute cell count of CD4^+^ helper T cells and CD8^+^ cytotoxic T cells at different time points after SCT. See also Figure S1 in Supplementary Material for a representation of absolute numbers for predicted values for a 10-year-old child. ns, not significant; *P < 0.05; **P < 0.01; ***P < 0.001.

We fitted a joint model for OS and each of the following leukocyte subsets: overall leukocytes, lymphocytes, CD14^+^ monocytes, CD3^+^ T cells, CD3^+^CD4^+^ helper T cells, CD3^+^CD8^+^ cytotoxic T cells, CD3^−^CD56^+^ NK cells, and CD19^+^ B cells, as shown in Figure 2. Such modeling allowed a comparison of leukocyte subset recovery after PBSC_CD34sel_ and PBSC_CD3/CD19dep_ transplantations. Tables 2 and 3 present the respective immune recovery prognosis values of our models showing the predicted percent values (% of norm values) and their 95% confidence intervals on days +30, +60, +90, and +180 and +365 post haplo-SCT for children in CR after SCT. As an example, predicted values for the absolute numbers with 95% CI are shown for a 10-year-old child (Figure S1 and Tables S2A,B in Supplementary Material).

**Table 2:** Predicted cells immune reconstitution (% norm values) in patients who received myeloablative conditioning regimen.

Cells	Day + 30		Day + 60		Day + 90		Day + 180		Day + 365	
	CD34sel	CD3/CD19dep	CD34sel	CD3/CD19dep	CD34sel	CD3/CD19dep	CD34sel	CD3/CD19dep	CD34sel	CD3/CD19dep
Leukocytes	34.22	39.98	52.49	47.05	64.26	50.23	66.80	49.21	75.69	56.14
	(25.94–45.15)	(34.91–45.79)	(38.82–70.98)	(41.29–53.60)	(47.11–87.64)	(44.15–57.14)	(53.09–84.04)	(43.78–55.31)	(61.47–93.19)	(49.47–63.72)
Lymphocytes	15.69	12.75	35.20	21.93	42.23	25.75	31.67	25.05	36.43	36.18
	(12.39–19.86)	(10.80–15.04)	(28.66–42.23)	(18.68–25.75)	(34.98–50.99)	(22.13–29.98)	(27.21–36.85)	(21.72–28.90)	(31.11–42.67)	(31.36–41.74)
Monocytes	64.81	86.29	68.52	71.44	68.99	62.25	64.91	51.04	72.76	54.14
	(48.21–87.14)	(72.61–102.56)	(53.69–87.44)	(60.32–84.61)	(53.85–88.39)	(52.62–73.64)	(54.22–77.71)	(44.01–59.19)	(60.36–87.71)	(46.34–63.26)
CD3^+^	1.18	1.15	5.23	4.90	9.13	7.98	21.05	16.35	36.98	30.94
	(0.82–1.69)	(0.91–1.45)	(3.76–7.30)	(3.91–6.15)	(6.98–11.94)	(6.54–9.73)	(16.80–26.38)	(13.48–19.82)	(29.83–45.85)	(25.31–37.81)
CD4^+^	0.91	1.32	2.32	3.22	3.10	4.44	5.15	7.68	9.27	14.08
	(0.72–1.16)	(0.94–1.86)	(1.90–2.85)	(2.46–4.21)	(2.59–3,72)	(3.63–5.44)	(4.29–6.18)	(6.18–9.54)	(7.79–11.04)	(11.81–16.77)
CD8^+^	2.24	1.52	7.13	7.22	11.91	15.85	22.13	26.30	51.33	37.96
	(1.82–2.76)	(1.0–2.33)	(6.17–8.24)	(5.48–9.52)	(10.47–13.56)	(12.80–19.63)	(18.57–25.01)	(21.21–32.62)	(45.58–57.80)	(31.36–45.94)
CD3^−^CD56^+^	88.93	65.10	145.31	78.72	85.19	58.80	47.80	44.25	47.66	46.51
	(68.93–115.23)	(53.59–79.07)	(114.59–184.27)	(65.0–94.34)	(72.35–100.31)	(49.56–69.77)	(40.37–56.60)	(37.23–52.59)	(41.79–54.35)	(39.65–54.55)
CD19^+^ B	2.82	1.71	10.57	5.09	21.46	8.59	24.52	7.30	25.49	9.35
	(1.86–4.26)	(1.40–2.09)	(7.12–15.70)	(4.23–6.13)	(14.45–31.89)	(7.10–10.40)	(18.55–32.40)	(6.16–8.65)	(18.98–34.24)	(7.77–11.26)

*The table shows the predicted percent values (% of the norm values) arise from our joint model with 95% confidence interval at +30, +60, +90, +120, and +365 days after transplantation. The values were obtained for children in complete remission (CR) at time point of haplo-SCT. The percent values represent the fraction with respect to the predicted age-matched norm values by Huenecke et al. (27). See also Table S2A in Supplementary Material for a representation of absolute numbers for predicted values for a 10-year-old child*.

#### Impact of Both Conditioning Regimen and Graft on IR of Patients in CR

The majority of the 29 patients transplanted in CR with a CD3/CD19-depleted graft received reduced intensity conditioning, while the majority of the 11 patients transplanted in CR with a CD34-selected graft underwent full myeloablative conditioning (see Figure 1 for full details). To separate the influence of conditioning regimen and graft manipulation, all four scenarios (CR-MAC PBSC_CD34sel_, CR-MAC PBSC_CD3/CD19dep_, CR-RIC PBSC_CD34sel_, and CR-RIC PBSC_CD3/CD19dep_) were compared.

The increase in overall leukocytes was similar in the RIC and MAC patients during the first year (Figure 2A). However, within the same conditioning regimen group, faster leukocyte proliferation was found in patients with PBSC_CD34sel_ grafts than in patients with PBSC_CD3/CD19dep_ grafts from the 6 months after SCT. Previously, the leukocyte populations were comparable between the two groups with graft manipulation.

The overall lymphocytes in patients receiving PBSC_CD34sel_ grafts differed from those with PBSC_CD3/CD19dep_ grafts, also showing a faster recovery in the early phase until the sixth month after haplo-SCT (Figure 2B, *P* = 0.036). This was mainly due to a faster reconstitution of NK cells and B cells in patients receiving PBSC_CD34sel_ vs. PBSC_CD3/CD19dep_ grafts. Afterward, the course of lymphocyte values was comparable in both groups. Concerning the conditioning regimen, we observed a faster expansion of the overall lymphocytes in the RIC group than in the MAC group. At the end of the first year, the RIC and MAC groups reached approximately 42 and 36% of the normal reference value, respectively.

Monocyte expansion was similar in RIC and MAC recipients, but the number of cells did not exhibit significant differences with respect to graft purification. For the overall monocytes, we observed a rapid IR, with approximately 80% of their age norm value already at 30 days after SCT (Figure 2C).

CD3^+^ T cells, CD3^+^CD4^+^ helper T cells, and the CD3^+^CD8^+^ cytotoxic T cells recovered significantly quicker in the patients receiving the RIC regimen than in those patients receiving MAC regimen (Figures 2D–F, *P* < 0.001). In the RIC group, the frequency values were nearly 1.5 times higher at each time point compared to the respective kind of manipulation receiving the MAC regimen during the first year post haplo-SCT. No significant differences were detected in the recovery of CD3^+^ T cells and the CD3^+^CD8^+^ cytotoxic T cells owing to the kind of graft purification, PBSC_CD34sel_, and PBSC_CD3/CD19dep_, among patients undergoing the same regimen of conditioning. The CD3^+^ T cells increased from 1% of the reference values at day 30 to 37% at the end of the first year and from 1 to 31% for PBSC_CD34sel_ and PBSC_CD3/CD19dep_, respectively, with both groups receiving MAC (Table 2). In patients with the RIC regimen, the CD3^+^ T cells increased from 1.7% of the reference values at day 30 to 54% at the end of the first year and from 1 to 45% for PBSC_CD34sel_ and PBSC_CD19dep_, respectively (Table 3).

**Table 3:** Predicted cells immune reconstitution (% norm) in patients who received reduce-intensity conditioning regimen.

Cells	Day + 30		Day + 60		Day + 90		Day + 180		Day + 365	
	CD34sel	CD3/CD19dep	CD34sel	CD3/CD19dep	CD34sel	CD3/CD19dep	CD34sel	CD3/CD19dep	CD34sel	CD3/CD19dep
Leukocytes	37.24	43.50	57.12	51.19	69.92	54.65	72.68	53.54	82.35	61.09
	(28.97–47.87)	(38.83–48.73)	(43.79–74.50)	(45.61–57.46)	(53.28–91.75)	(48.60–61.45)	(60.20–87.75)	(48.42–59.21)	(69.19–98.02)	(55.66–67.05)
Lymphocytes	18.39	14.94	41.27	25.71	49.52	30.20	37.13	29.38	42.72	42.42
	(14.35–23.57)	(12.68–17.62)	(32.9–51.77)	(21.8–30.34)	(40.17–61.03)	(25.72–35.45)	(31.71–43.48)	(25.17–34.28)	(36.15–50.48)	(36.78–48.94)
Monocytes	62.16	82.76	65.72	68.52	66.17	59.70	62.26	48.95	69.79	51.93
	(46.53–83.05)	(70.60–97.03)	(51.35–84.11)	(58.33–80.49)	(51.42–85.15)	(50.74–70.25)	(52.58–73.71)	(42.50–56.38)	(58.19–83.69)	(43.35–59.46)
CD3^+^	1.72	1.68	7.65	7.17	13.35	11.66	30.77	23.90	54.07	45.23
	(1.2–2.45)	(1.4–2.01)	(5.49–10.66)	(5.97–8.61)	(10.24–17.40)	(10.04–13.55)	(24.79–38.19)	(20.74–27.54)	(43.42–67.32)	(39.53–51.75)
CD4^+^	1.45	2.10	3.70	5.13	4.94	7.07	8.20	12.23	14.77	22.41
	(1.18–1.79)	(1.49–2.98)	(3.11–4.41)	(3.91–6.72)	(4.25–5.74)	(5.78–8.64)	(7.06–9.53)	(9.86–15.16)	(13.01–16.76)	(18.57–27.05)
CD8^+^	4.28	2.91	13.62	13.80	22.76	30.28	42.26	50.25	98.05	72.51
	(3.29–5.56)	(1.91–4.43)	(11.07–16.77)	(10.51–18.12)	(18.76–27.62)	(24.53–37.37)	(34.92–51.16)	(40.89–61.75)	(80.66–119.18)	(61.50–85.49)
CD3^−^CD56^+^	97.35	71.26	159.07	86.18	93.26	64.37	52.33	48.44	52.17	50.91
	(74.37–127.42)	(60.81–83.50)	(124.26–203.63)	(73.38–101.21)	(78.73–110.5)	(55.62–74.5)	(44.01–62.21)	(41.68–56.29)	(44.83–60.72)	(45.37–57.13)
CD19^+^ B	3.24	1.97	12.15	5.85	24.68	9.88	28.18	8.40	29.31	10.75
	(2.18–4.81)	(1.44–2.68)	(8.41–17.55)	(4.35–7.87)	(17.12–35.56)	(7.36–13.27)	(21.97–36.16)	(6.39–11.03)	(22.38–38.37)	(8.07–14.32)

*The table shows the predicted percent values (% of the norm values) arise from our joint model with 95% confidence interval at +30, +60, +90, +120, and +365 days after transplantation. The values were obtained for children in complete remission (CR) at time point of haplo-SCT. The percent values represent the fraction with respect the predicted age-matched norm values by Huenecke et al. (27). See also Table S2B in Supplementary Material for a representation of absolute numbers for predicted values for a 10-year-old child*.

In contrast, the CD3^+^CD4^+^ helper T cells recovered significantly delayed in patients receiving PBSC_CD34sel_ grafts than in those transplanted with PBSC_CD3/CD19dep_ grafts from approximately 6th to 24th months after transplantation for both the RIC and MAC regimens (*P* < 0.01).

In both the RIC and MAC groups, the ratio of CD4^+^/CD8^+^ trended downward because the cytotoxic T cells recovered faster than the helper T cells (Figure 2I).

As expected, *in vivo* NK expansion post haplo-SCT was very rapid (Figure 2G). No differences in the NK cell recovery were found between the RIC and MAC groups. Interestingly, in the first 4 months, NK cell recovery was faster in patients receiving PBSC_CD34sel_ grafts than in those transplanted with a PBSC_CD3/CD19dep_ graft (*P* = 0.002). Afterward, the *in vivo* NK cell proliferation was very similar between the patient groups.

Recovery of CD19^+^ B cells showed no differences in the IR regarding the conditioning regimen (Figure 2H). With regard to graft manipulation, B-cell recovery was faster in the PBSC_CD34sel_ patient group than in the PBSC_CD3/CD19dep_ group (*P* < 0.001). This difference was very pronounced in the first year after haplo-SCT. At the end of the first year, the B cells in the group PBSC_CD34sel_ with RIC had already achieved 30% of the age-matched reference values. In contrast, in the PBSC_CD3/CD19dep_ with MAC group, the B cells had only reached 9% of the age reference values.

### Clinical Outcome and Survival

The medians (range) OS follow-up time were 149 (72–160.3) and 101 (44–138) months in patients receiving PBSC_CD34se_ and PBSC_CD3/CD19dep_, respectively.

The incidence of aGvHD grades I and II was 45% (5/11) in patients with PBSC_CD34sel_ grafts and 48% (14/29) in patients with PBSC_CD3/CD19dep_ grafts. The incidence of aGvHD grades III and IV was 18% (2/11) and 14% (4/29) in the cohorts with PBSC_CD34sel_ vs. PBSC_CD3/CD19dep_ grafts, respectively. The incidence of aGvHD did not significantly differ between both groups. Table 4 presents details of the clinical outcome.

**Table 4:** Clinical outcome.

	CD34 sel	CD3/CD19 dep	*P*
		
	*n* = 11	*n* = 29	
Engraftment			
Neutrophils engraftment			0.054
Median (range) days	17 (11–22)	12 (10–24)	
Graft vs. host disease			
Acute GvHD			1
Without	4	11	
Grade I/II	2/3	12/2	
Grade III/IV	1/1	3/1	
Site of aGVHD			
Skin	4	13	
Skin, GI	1	2	
Skin, GI, liver	–	1	
Skin, GI, lung	1	2	
No data	1	0	
Chronic GvHD	3	2	0.117
Cause of death			
Relapse	4	9	
Treatment-related mortality	3	5	

*GvHD, graft vs. host disease; GI, gastrointestinal. P-value was calculated using Fisher-Exact test or Mann–Whitney–Wilcoxon test*.

**Figure 3:**
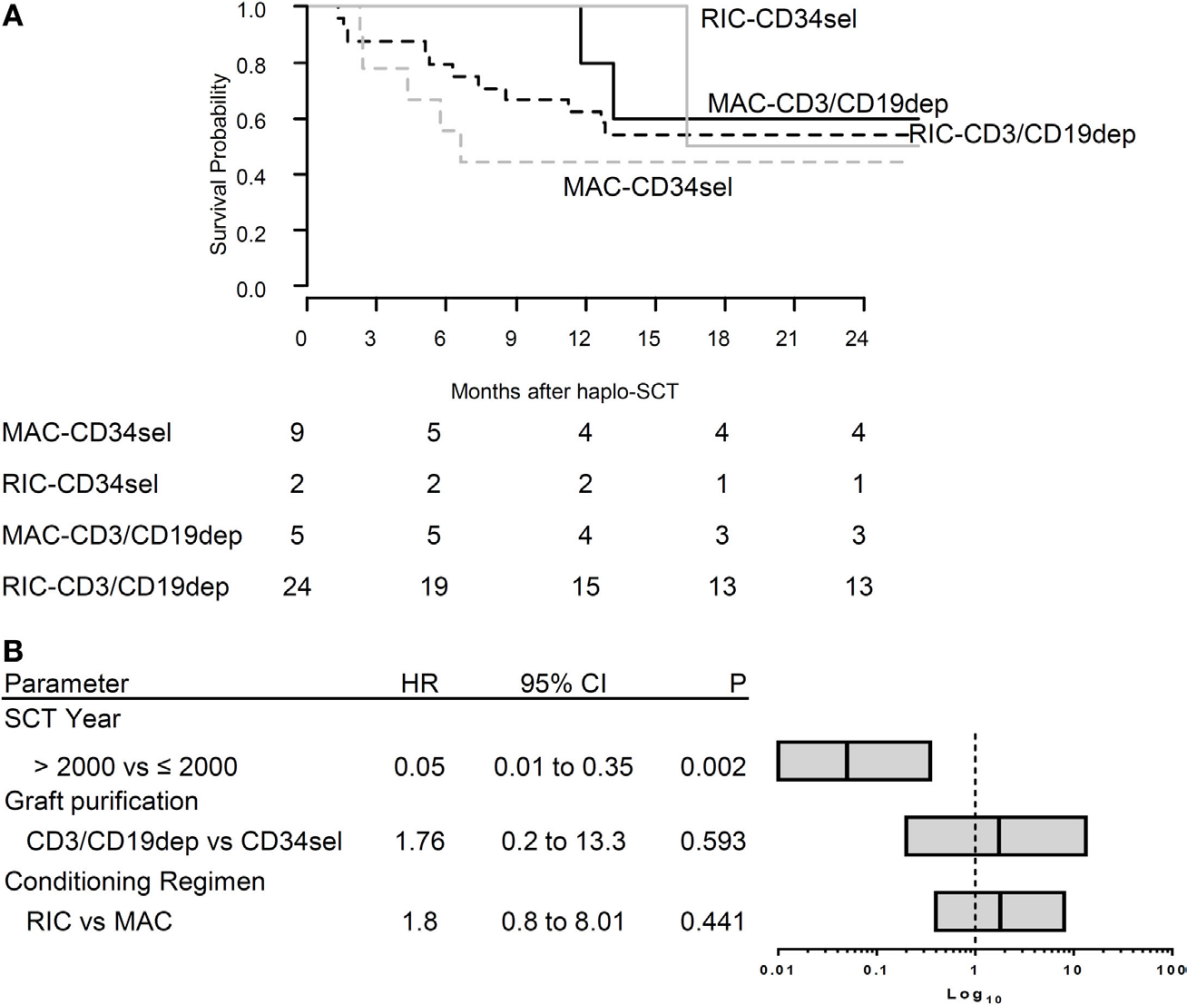
**(A)** Survival overall survival (OS). The Kaplan–Meier curve of OS for CR patients according to type of graft (CD34sel, CD3/CD19dep) combined with conditioning regimen (MAC, RIC). **(B)** Multivariable analysis of clinical characteristics affecting OS. The plot shows the hazard ratio (HR) of multivariable Cox regression. The stem cell transplantation year was significant correlated with OS. The bar in each box represents the HR, the box the corresponding 95% confidence interval. The vertical line represents HR = 1 for reference.

Overall, 21/40 patients died. The main cause of death was relapse (*n* = 13), followed by non-relapse mortality (NRM, *n* = 8). The Kaplan–Meier curves for OS were compared between the different groups (Figure 3A). The log rank test was not significant for differences between the PBSC_CD34sel_ and the PBSC_CD3/CD19dep_ groups (Table S3 in Supplementary Material). Likewise, there were no significant differences in the causes of death between the PBSC_CD34sel_ and the PBSC_CD3/CD19dep_ patient groups.

Using standard Cox regression, we analyzed the association between transplant-associated factors and the probability of OS without considering the evolution of IR after haplo-SCT. In the univariable analysis, age, gender, diagnosis, TBI (yes/no), conditioning regimen, graft purification, graft composition, and engraftment day were not associated with OS. In the multivariable regression considering graft purification, conditioning regimen and year of transplantation (≤2001 vs. >2001), only the year of transplantation (*P* = 0.002) significantly influenced OS. The conditioning regimen had no significant impact on OS in our cohort (*P* = 0.441) (Figure 3B).

## Discussion

To date, there are ongoing discussions about optimization of graft composition in haplo-SCT in order to reach a sufficient IR with an additional goal to improve the GVL/GVT effect and, finally, the survival of the patients. Various factors affecting IR after SCT, especially haplo-SCT (5–10), have been identified, such as age, diagnosis, stage of disease, remission status at transplantation, conditioning regimen, HLA-typing, and graft manipulation (6, 40–42).

Investigation of IR post SCT, especially haplo-SCT, has been done in single-center studies, mostly in heterogeneous patient groups using univariate analyses. Here, we compared the dynamics of IR in patients receiving PBSC_CD34sel_ vs. PBSC_CD3/CD19dep_ when used for haplo-SCT in a homogeneous pediatric patient group with acute leukemia from a retrospective multicenter study. To investigate the complexity of various factors on IR, we were able to develop joint models for longitudinal and time-to-event data. To our knowledge, this is the first time that the influence of multivariable clinical factors, including RIC, MAC, and age-related dependence of leukocytes and their subpopulations, has been evaluated in detail in haplo-SCT from pediatric patients suffering leukemia.

Surprisingly, IR for T cells did not differ between patients receiving PBSC_CD34sel_ or PBSC_CD3/CD19dep_ grafts with the exception of CD4^+^ helper T cells, which showed much slower recovery after transplantation using a PBSC_CD34selp_ graft. NK and B cells had a faster reconstitution after transfusion of the PBSC_CD34sel_ graft. In this respect, it has to be noted that direct comparisons of IR after transplantation with PBSC_CD3/CD19dep_ compared with PBSC_CD34sel_ grafts have to be handled with caution because patients receiving the latter normally do not need immunosuppressive therapy. However, the most important factor for T-cell and T subsets recovery was the conditioning regimen. For patients in CR receiving either PBSC_CD34sel_ or PBSC_CD3/CD19dep_ grafts, IR was much faster after the RIC regimen than after the MAC regimen. However, it has to be mentioned that the main limitations of our study are its retrospective nature and the small number of patients, although it is a multicenter study.

Comparison of our results with other haploidentical pediatric studies in the literature showed that most of the other studies about IR were using very heterogeneous patient groups (43–45) with only some of them treated with haplo-SCT (2, 46–52) or analyzed IR including patients with PR/NR (45, 53). Other investigations are not comparable with our study due to the administration of fixed addback (54, 55), or reported about other malignant (56) or nonmalignant diseases (57).

A high dose of CD34 cells is required in the haploidentical setting for a successful transplant and therefore rapid and sustained engraftment (58). Despite the differences in the CD34 doses between the CD34 selected group and the CD3/CD19-depleted group, no differences in the neutrophil engraftment or aGVHD was found.

In contrast to our findings that the IR of the CD3^+^ T cells and CD8^+^ cytotoxic T cells was comparable between recipients receiving haplo-PBSC_CD3/CD19dep_ and haplo-PBSC_CD34sel_ if both groups received the same conditioning regimen, other researchers reported slower expansion of T cells, CD4^+^, and CD8^+^ cells in recipients receiving mismatch/haplo-PBSC_CD34sel_ than in recipients receiving haplo-PBSC_CD3/CD19dep_ (41, 49). A possible reason for this discrepancy may be that the mentioned studies did not differentiate between the influences of the graft and the conditioning on IR. The majority of patients received PBSC_CD34sel_ combined with the MAC regimen or PBSC_CD3/CD19dep_ combined with the RIC regimen. When we compared the IR between the PBSC_CD34sel_ vs. PBSC_CD3/CD19dep_ without considering conditioning, our results were consistent with these previous findings (data not shown).

In our study, there was no difference in the CD3^+^CD4^+^ helper T-cell recovery between patients receiving depleted or selected grafts until the sixth month after haplo-SCT. Therefore, it seems unlikely that better expansion of helper T cells at late time points is explained by residual T cells in the CD3/CD19-depleted graft. However, after the sixth month, the CD3^+^CD4^+^ helper T cells recovered significantly faster in patients receiving CD3/CD19-depleted grafts than in those receiving CD34-selected grafts, regardless of the type of conditioning. This could also be the effect of other therapeutic agents applied combined with the CD3/CD19-depleted graft.

It is well known that RIC facilitates rapid reconstitution of T cells and its subsets in PBSC transplantation. However, the RIC method is often not strong enough to eradicate the malignant cells, and it is associated with a higher incidence of mixed chimerism (59), higher relapse, and mortality risk (60). After SCT, non-remission rates found in studies with patients who received haplo-PBSC_CD3/CD19dep_ combined with an RIC regimen were 50.2% (61), 68% (45), and 57% (53), but note that 31 of 61 adult patients did not achieve CR in the first study, 22 of 26 analyzed childhood patients in the second study suffered from malignancies, and 43% of patients from the third study had active disease at the time of transplantation.

Surprisingly, we observed higher expansion of NK cells in the early phase of SCT in patients receiving a PBSC_CD34sel_ graft than in those patients who received a PBSC_CD3/CD19dep_ graft, despite the fact that PBSC_CD3/CD19dep_ grafts contain considerably higher numbers of NK cells. Furthermore, we did not find any differences between the immune recoveries of NK cells when comparing positive selection or negative depletion with conditioning preparative treatments MAC vs. RIC. Our results about NK expansion in the PBSC_CD34sel_ combined with MAC are in conformity with the study from Lang et al. (50). In that trial (*n* = 47), most of the pediatric patients suffered from acute leukemia (*n* = 37; 78%) were transplanted from a haploidentical donor (*n* = 29; 62%) and underwent haplo-PBSC_CD34sel_ after MAC. The NK cells shown a pick with a median of 300 cells/μl reported by Lang et al, we found a peak with a median of 323 cells/μl as shown exemplarily for a 10-year-old recipient (Table S2A in Supplementary Material).

Pfeiffer et al. reported a comparable NK cell reconstitution between patients receiving a haplo-PBSC_CD3/CD19dep_ graft with RIC and those patients receiving mismatch-PBSC_CD34sel_ grafts with MAC in pediatric patients. They found that only at day +14 was there a significant difference advantageous for PBSC_CD3/CD19dep_ recipients (45). However, the HLA-disparity and related donors were different between the PBSC_CD34sel_ groups in our and the mentioned study, and the latter included heterogeneous diseases with 64% cases of acute leukemia in the mismatch-PBSC_CD34sel_ group and only 42% cases of acute leukemia in the haplo-PBSC_CD3/CD19dep_ group. Additionally, Pfeiffer reported a rate of 42% with active diseases (PR/NR) prior to SCT in the CD3/CD19-depleted group, while our study included patients in CR only.

Significantly higher NK cell proliferation in patients receiving PBSC_CD3/CD19dep_ grafts than in patients undergoing PBSC_CD34sel_ (both groups combined with RIC) was presented by Gonzalez-Vicent et al. (51). However, in this investigation, 74% of patients with PBSC_CD34sel_ had a matched donor, while all patients with PBSC_CD3/CD19dep_ received a transplant from mismatched donors.

Alternatively, unmanipulated blood and marrow transplantation is another feasible option in haploidentical transplantations. Marek et al. (62) examined lymphocyte recovery of haploidentical *in vivo* (*n* = 19) vs. *in vitro* T-cell depletion (*n* = 53, 89% haploidentical donors, 81% acute leukemia, 76% children). The *in vitro* T-cell depletion was mostly PBSC_CD34sel_ (94%) with the MAC regimen (78%). They reported faster IR not only of the NK cells but also of all lymphocyte subpopulations in patients receiving grafts with *in vitro* T-cell depletion than observed in our results. Nevertheless, they used a MAC regimen based on cyclophosphamide + TBI instead of VP16 + TBI as in our study.

There are no studies comparing B-cell recovery in pediatric patients who received RIC vs. MAC regimens after haplo-SCT. However, in ALL childhood patients, the chemotherapy strongly affects B cells (63). In adult studies, there is no consensus about this point (7, 64–67).

Concerning the graft manipulation technique, similar to our results, Bethge et al. (20) reported faster B-cell expansion in adult patients receiving haplo-PBSC_CD34sel_ than in those who received haplo-PBSC_CD3/CD19dep_ grafts. The influence of serotherapy on the IR especially on the B cells is reported in several studies (68–70). In our study, the size of the cohort did not allow the inclusion of this factor in our model.

Booth et al. reported the unpublished observations from Lawson and Darbyshire (71). They found no significant difference between the CD34 selection compared to CD3/CD19 depletion in terms of engraftment, IR, viral infections, or non-relapse mortality.

## Conclusion

We analyzed a homogeneous pediatric group suffering from high-risk acute leukemia in CR, who underwent *in vitro* haplo-PBSCT in a retrospective study. A limitation of our study is the small number of patients, although it is a multicenter study.

Comparing transplantation with CD34-positive selected and CD3/CD19-depleted grafts patients did not show marked differences in the IR, while differences were stronger between the conditioning regimens. For further optimization of the treatment of acute childhood leukemia, conditioning regimen, graft purification, and their interplay should always be considered, particularly for novel techniques of graft manipulation and engineering, such as TCR alpha/beta depletion and *in vivo* T-cell depletion, as well as genetically modified T-cell products.

## Ethics Statement

Informed consent was obtained from all patients/parents, and the retrospective study was approved by the respective local ethic committee (Frankfurt EK 499/16, 50/07), Würzburg (EK 133/04) and has been included in part in the registered studies for both, transplantation with CD3/CD19 depleted grafts (EudraCT-No.: 2006-000393-76) as well as the ALL-SCT study (register No. 3352).

## Author Contributions

Study design and wrote the manuscript: UK, EH, and ES-M. Provided clinical data: ME, AS, and PB. Analyzed the data: RE, MB, and SH. Coordinated the research: UK and EH. Organized and data collection: ES-M, MS, and MB. Performed statistical analyses: ES-M. Revised the manuscript: UK, EH, SH, MB, ME, and PB. Supervised the research: EH, UK, and TK. All authors read and approved the final manuscript.

## Conflict of Interest Statement

The authors declare that the research was conducted in the absence of any commercial or financial relationships that could be construed as a potential conflict of interest.

## References

[1] Devine SM Adkins DR Khoury H Brown RA Vij R Blum W Recent advances in allogeneic hematopoietic stem-cell transplantation. J Lab Clin Med (2003) 141:7–32. 10.1067/mlc.2003.5 12518165

[2] Klingebiel T Handgretinger R Lang P Bader P Niethammer D. Haploidentical transplantation for acute lymphoblastic leukemia in childhood. Blood Rev (2004) 18:181–92. 10.1016/S0268-960X(03)00063-8 15183902

[3] Barrett AJ Horowitz MM Pollock BH Zhang MJ Bortin MM Buchanan GR Bone marrow transplants from HLA-identical siblings as compared with chemotherapy for children with acute lymphoblastic leukemia in a second remission. N Engl J Med (1994) 331:1253–8. 10.1056/NEJM199411103311902 7935682

[4] Horowitz MM Gale RP Sondel PM Goldman JM Kersey J Kolb HJ Graft-versus-leukemia reactions after bone marrow transplantation. Blood (1990) 75:555–62. 2297567

[5] Storek J Dawson MA Storer B Stevens-Ayers T Maloney DG Marr KA Immune reconstitution after allogeneic marrow transplantation compared with blood stem cell transplantation. Blood (2001) 97:3380–9. 10.1182/blood.V97.11.3380 11369627

[6] Kalwak K Gorczyńska E Toporski J Turkiewicz D Slociak M Ussowicz M Immune reconstitution after haematopoietic cell transplantation in children: immunophenotype analysis with regard to factors affecting the speed of recovery. Br J Haematol (2002) 118:74–89. 10.1046/j.1365-2141.2002.03560.x 12100130

[7] Morecki S Gelfand Y Nagler A Or R Naparstek E Varadi G Immune reconstitution following allogeneic stem cell transplantation in recipients conditioned by low intensity vs myeloablative regimen. Bone Marrow Transplant (2001) 28:243–9. 10.1038/sj.bmt.1703118 11535991

[8] Koehl U Bochennek K Zimmermann SY Lehrnbecher T Sörensen J Esser R Immune recovery in children undergoing allogeneic stem cell transplantation: absolute CD8+ CD3+ count reconstitution is associated with survival. Bone Marrow Transplant (2007) 39:269–78. 10.1038/sj.bmt.1705584 17311085

[9] Koenig M Huenecke S Salzmann-Manrique E Esser R Quaritsch R Steinhilber D Multivariate analyses of immune reconstitution in children after allo-SCT: risk-estimation based on age-matched leukocyte sub-populations. Bone Marrow Transplant (2010) 45:613–21. 10.1038/bmt.2009.204 19701252

[10] Schwinger W Weber-Mzell D Zois B Rojacher T Benesch M Lackner H Immune reconstitution after purified autologous and allogeneic blood stem cell transplantation compared with unmanipulated bone marrow transplantation in children. Br J Haematol (2006) 135:76–84. 10.1111/j.1365-2141.2006.06244.x 16925797

[11] Powles R Singhal S Treleaven J Kulkarni S Horton C Mehta J. Identification of patients who may benefit from prophylactic immunotherapy after bone marrow transplantation for acute myeloid leukemia on the basis of lymphocyte recovery early after transplantation. Blood (1998) 91:3481–6. 9558408

[12] Daley JP Rozans MK Smith BR Burakoff SJ Rappeport JM Miller RA. Retarded recovery of functional T cell frequencies in T cell-depleted bone marrow transplant recipients. Blood (1987) 70:960–4. 3307954

[13] Einsele H Ehninger G Steidle M Fischer I Bihler S Gerneth F Lymphocytopenia as an unfavorable prognostic factor in patients with cytomegalovirus infection after bone marrow transplantation. Blood (1993) 82:1672–8. 8395913

[14] Castillo N García-Cadenas I Barba P Canals C Díaz-Heredia C Martino R Early and long-term impaired T lymphocyte immune reconstitution after cord blood transplantation with antithymocyte globulin. Biol Blood Marrow Transplant (2017) 23(3):491–7. 10.1016/j.bbmt.2016.11.014 27888015

[15] Aversa F Velardi A Tabilio A Reisner Y Martelli MF. Haploidentical stem cell transplantation in leukemia. Blood Rev (2001) 15:111–9. 10.1054/blre.2001.0157 11735159

[16] Bethge WA Haegele M Faul C Lang P Schumm M Bornhauser M Haploidentical allogeneic hematopoietic cell transplantation in adults with reduced-intensity conditioning and CD3/CD19 depletion: fast engraftment and low toxicity. Exp Hematol (2006) 34:1746–52. 10.1016/j.exphem.2006.08.009 17157172

[17] Lang P Greil J Bader P Handgretinger R Klingebiel T Schumm M Long-term outcome after haploidentical stem cell transplantation in children. Blood Cells Mol Dis (2004) 33:281–7. 10.1016/j.bcmd.2004.08.017 15528145

[18] Ball LM Egeler RM Acute GvHD: pathogenesis and classification. Bone Marrow Transplant (2008) 41(Suppl 2):64 10.1038/bmt.2008.56 18545246

[19] Ferrara JLM Levine JE Reddy P Holler E. Graft-versus-host disease. Lancet (2009) 373:1550–61. 10.1016/S0140-6736(09)60237-3 19282026 PMC2735047

[20] Bethge WA Faul C Bornhäuser M Stuhler G Beelen DW Lang P Haploidentical allogeneic hematopoietic cell transplantation in adults using CD3/CD19 depletion and reduced intensity conditioning: an update. Blood Cells Mol Dis (2008) 40:13–9. 10.1016/j.bcmd.2007.07.001 17869547

[21] Federmann B Hägele M Pfeiffer M Wirths S Schumm M Faul C Immune reconstitution after haploidentical hematopoietic cell transplantation: impact of reduced intensity conditioning and CD3/CD19 depleted grafts. Leukemia (2011) 25:121–9. 10.1038/leu.2010.235 20944677

[22] Handgretinger R Negative depletion of CD3(+) and TcRαβ(+) T cells. Curr Opin Hematol (2012) 19:434–9. 10.1097/MOH.0b013e3283582340 22914586

[23] Huenecke S Bremm M Cappel C Esser R Quaiser A Bonig H Optimization of individualized graft composition: CD3/CD19 depletion combined with CD34 selection for haploidentical transplantation. Transfusion (2016) 56:2336–45. 10.1111/trf.13694 27346253

[24] Gordon PR Leimig T Mueller I Babarin-Dorner A Holladay MA Houston J A large-scale method for T cell depletion: towards graft engineering of mobilized peripheral blood stem cells. Bone Marrow Transplant (2002) 30:69–74. 10.1038/sj.bmt.1703619 12132044

[25] Koehl U Bochennek K Esser R Brinkmann A Quaritsch R Becker M ISHAGE-based single-platform flowcytometric analysis for measurement of absolute viable T cells in fresh or cryopreserved products: CD34/CD133 selected or CD3/CD19 depleted stem cells, DLI and purified CD56+CD3- NK cells. Int J Hematol (2008) 87:98–105. 10.1007/s12185-007-0018-7 18224422

[26] Przepiorka D Weisdorf D Martin P Klingemann HG Beatty P Hows J 1994 consensus conference on acute GVHD grading. Bone Marrow Transplant (1995) 15:825–8. 7581076

[27] Huenecke S Behl M Fadler C Zimmermann SY Bochennek K Tramsen L Age-matched lymphocyte subpopulation reference values in childhood and adolescence: application of exponential regression analysis. Eur J Haematol (2008) 80:532–9. 10.1111/j.1600-0609.2008.01052.x 18284628

[28] Comans-Bitter WM de Groot R van den Beemd R Neijens HJ Hop WC Groeneveld K Immunophenotyping of blood lymphocytes in childhood. Reference values for lymphocyte subpopulations. J Pediatr (1997) 130:388–93. 10.1016/S0022-3476(97)70200-2 9063413

[29] van Gent R van Tilburg CM Nibbelke EE Otto SA Gaiser JF Janssens-Korpela PL Refined characterization and reference values of the pediatric T- and B-cell compartments. Clin Immunol (2009) 133:95–107. 10.1016/j.clim.2009.05.020 19586803

[30] Shearer WT Rosenblatt HM Gelman RS Oyomopito R Plaeger S Stiehm ER Lymphocyte subsets in healthy children from birth through 18 years of age: the pediatric AIDS clinical trials group P1009 study. J Allergy Clin Immunol (2003) 112:973–80. 10.1016/j.jaci.2003.07.003 14610491

[31] Tosato F Bucciol G Pantano G Putti MC Sanzari MC Basso G Lymphocytes subsets reference values in childhood. Cytometry A (2015) 87:81–5. 10.1002/cyto.a.22520 25132325

[32] Schatorjé EJH Gemen EFA Driessen GJA Leuvenink J van Hout RWNM van der Burg M Age-matched reference values for B-lymphocyte subpopulations and CVID classifications in children. Scand J Immunol (2011) 74:502–10. 10.1111/j.1365-3083.2011.02609.x 21815909

[33] Schatorjé EJH Gemen EFA Driessen GJA Leuvenink J van Hout RWNM de Vries E. Paediatric reference values for the peripheral T cell compartment. Scand J Immunol (2012) 75:436–44. 10.1111/j.1365-3083.2012.02671.x 22420532

[34] Duchamp M Sterlin D Diabate A Uring-Lambert B Guérin-El Khourouj V Le Mauff B B-cell subpopulations in children: National reference values. Immun Inflamm Dis (2014) 2:131–40. 10.1002/iid3.26 25505547 PMC4257758

[35] Wiener D Shah S Malone J Lowell N Lowitt S Rowlands DT. Multiparametric analysis of peripheral blood in the normal pediatric population by flow cytometry. J Clin Lab Anal (1990) 4:175–9. 10.1002/jcla.1860040305 2352053

[36] Henderson R Diggle P Dobson A. Joint modelling of longitudinal measurements and event time data. Biostatistics (2000) 1:465–80. 10.1093/biostatistics/1.4.465 12933568

[37] Verbeke G Molenberghs G Linear Mixed Models for Longitudinal Data. New York: Springer (2009).

[38] Therneau TM Grambsch PM Modeling survival Data. Extending the Cox Model. New York: Springer (2000).

[39] Rizopoulos D Joint Models for Longitudinal and Time-to-Event Data. With Applications in R. Boca Raton: CRC Press (2012).

[40] Behringer D Bertz H Schmoor C Berger C Dwenger A Finke J. Quantitative lymphocyte subset reconstitution after allogeneic hematopoietic transplantation from matched related donors with CD34+ selected PBPC grafts unselected PBPC grafts or BM grafts. Bone Marrow Transplant (1999) 24:295–302. 10.1038/sj.bmt.1701889 10455369

[41] Lang P Handgretinger R. Haploidentical SCT in children: an update and future perspectives. Bone Marrow Transplant (2008) 42(Suppl 2):9. 10.1038/bmt.2008.285 18978746

[42] Marks DI Khattry N Cummins M Goulden N Green A Harvey J Haploidentical stem cell transplantation for children with acute leukaemia. Br J Haematol (2006) 134:196–201. 10.1111/j.1365-2141.2006.06140.x 16846478

[43] Eyrich M Lang P Lal S Bader P Handgretinger R Klingebiel T A prospective analysis of the pattern of immune reconstitution in a paediatric cohort following transplantation of positively selected human leucocyte antigen-disparate haematopoietic stem cells from parental donors. Br J Haematol (2001) 114:422–32. 10.1046/j.1365-2141.2001.02934.x 11529867

[44] Bader P Soerensen J Jarisch A Ponstingl E Krenn T Faber J Rapid immune recovery and low TRM in haploidentical stem cell transplantation in children and adolescence using CD3/CD19-depleted stem cells. Best Pract Res Clin Haematol (2011) 24:331–7. 10.1016/j.beha.2011.04.005 21925086

[45] Pfeiffer MM Feuchtinger T Teltschik H-M Schumm M Müller I Handgretinger R Reconstitution of natural killer cell receptors influences natural killer activity and relapse rate after haploidentical transplantation of T- and B-cell depleted grafts in children. Haematologica (2010) 95:1381–8. 10.3324/haematol.2009.021121 20145268 PMC2913088

[46] Handgretinger R Klingebiel T Lang P Schumm M Neu S Geiselhart A Megadose transplantation of purified peripheral blood CD34(+) progenitor cells from HLA-mismatched parental donors in children. Bone Marrow Transplant (2001) 27:777–83. 10.1038/sj.bmt.1702996 11477433

[47] Ortín M Raj R Kinning E Williams M Darbyshire PJ. Partially matched related donor peripheral blood progenitor cell transplantation in paediatric patients adding fludarabine and anti-lymphocyte gamma-globulin. Bone Marrow Transplant (2002) 30:359–66. 10.1038/sj.bmt.1703667 12235520

[48] Eyrich M Leiler C Croner T Lang P Schumm M Mascher B Impaired T-cell activation and cytokine productivity after transplantation of positively selected CD34+ allogeneic hematopoietic stem cells. Hematol J (2004) 5:329–40. 10.1038/sj.thj.6200397 15297850

[49] Lang P Schumm M Greil J Bader P Klingebiel T Müller I A comparison between three graft manipulation methods for haploidentical stem cell transplantation in pediatric patients: preliminary results of a pilot study. Klin Padiatr (2005) 217:334–8. 10.1055/s-2005-872529 16307419

[50] Lang P Pfeiffer M Teltschik HM Schlegel P Feuchtinger T Ebinger M Natural killer cell activity influences outcome after T cell depleted stem cell transplantation from matched unrelated and haploidentical donors. Best Pract Res Clin Haematol (2011) 24:403–11. 10.1016/j.beha.2011.04.009 21925093

[51] Gonzalez-Vicent M Perez A Abad L Sevilla J Ramirez M Diaz MA. Graft manipulation and reduced-intensity conditioning for allogeneic hematopoietic stem cell transplantation from mismatched unrelated and mismatched/haploidentical related donors in pediatric leukemia patients. J Pediatr Hematol Oncol (2010) 32:90. 10.1097/MPH.0b013e3181cf813c 20216238

[52] Pérez-Martínez A González-Vicent M Valentín J Aleo E Lassaletta A Sevilla J Early evaluation of immune reconstitution following allogeneic CD3/CD19-depleted grafts from alternative donors in childhood acute leukemia. Bone Marrow Transplant (2012) 47:1419–27. 10.1038/bmt.2012.43 22410752

[53] Lang P Teltschik HM Feuchtinger T Müller I Pfeiffer M Schumm M Transplantation of CD3/CD19 depleted allografts from haploidentical family donors in paediatric leukaemia. Br J Haematol (2014) 165:688–98. 10.1111/bjh.12810 24588540

[54] Bunin N Aplenc R Grupp S Pierson G Monos D. Unrelated donor or partially matched related donor peripheral stem cell transplant with CD34+ selection and CD3+ addback for pediatric patients with leukemias. Bone Marrow Transplant (2006) 37:143–9. 10.1038/sj.bmt.1705211 16284615

[55] Dvorak CC Gilman AL Horn B Oon C-Y Dunn EA Baxter-Lowe LA Haploidentical related-donor hematopoietic cell transplantation in children using megadoses of CliniMACs-selected CD34(+) cells and a fixed CD3(+) dose. Bone Marrow Transplant (2013) 48:508–13. 10.1038/bmt.2012.186 23178543

[56] Woodard P Cunningham JM Benaim E Chen X Hale G Horwitz E Effective donor lymphohematopoietic reconstitution after haploidentical CD34+-selected hematopoietic stem cell transplantation in children with refractory severe aplastic anemia. Bone Marrow Transplant (2004) 33:411–8. 10.1038/sj.bmt.1704358 14676782

[57] Lang P Klingebiel T Bader P Greil J Schumm M Schlegel PG Transplantation of highly purified peripheral-blood CD34+ progenitor cells from related and unrelated donors in children with nonmalignant diseases. Bone Marrow Transplant (2004) 33:25–32. 10.1038/sj.bmt.1704303 14704654

[58] Aversa F Prezioso L Manfra I Galaverna F Spolzino A Monti A. Immunity to infections after haploidentical hematopoietic stem cell transplantation. Mediterr J Hematol Infect Dis (2016) 8(1):e2016057. 10.4084/mjhid.2016.057 27872737 PMC5111540

[59] Perez A Gonzalez-Vicent M Ramirez M Sevilla J Madero L Diaz MA. Intentional induction of mixed haematopoietic chimerism as platform for cellular therapy after HLA-matched allogeneic stem cell transplantation in childhood leukaemia patients. Br J Haematol (2008) 140:340–3. 10.1111/j.1365-2141.2007.06911.x 18053071

[60] Peccatori J Ciceri F Allogeneic stem cell transplantation for acute myeloid leukemia. Haematologica (2010) 95:857–9. 10.3324/haematol.2010.023184 20513804 PMC2878779

[61] Federmann B Bornhauser M Meisner C Kordelas L Beelen DW Stuhler G Haploidentical allogeneic hematopoietic cell transplantation in adults using CD3/CD19 depletion and reduced intensity conditioning: a phase II study. Haematologica (2012) 97:1523–31. 10.3324/haematol.2011.059378 22491731 PMC3487553

[62] Marek A Stern M Chalandon Y Ansari M Ozsahin H Güngör T The impact of T-cell depletion techniques on the outcome after haploidentical hematopoietic SCT. Bone Marrow Transplant (2014) 49:55–61. 10.1038/bmt.2013.132 24037023

[63] Koskenvuo M Ekman I Saha E Salokannel E Matomäki J Ilonen J Immunological reconstitution in children after completing conventional chemotherapy of acute lymphoblastic leukemia is marked by impaired B-cell compartment. Pediatr Blood Cancer (2016) 63:1653–6. 10.1002/pbc.26047 27163649

[64] Maris M Boeckh M Storer B Dawson M White K Keng M Immunologic recovery after hematopoietic cell transplantation with nonmyeloablative conditioning. Exp Hematol (2003) 31:941–52. 10.1016/S0301-472X(03)00201-7 14550810

[65] Schulenburg A Fischer M Kalhs P Mitterbauer M Rabitsch W Greinix HT Immune recovery after conventional and non-myeloablative allogeneic stem cell transplantation. Leuk Lymphoma (2005) 46:1755–60. 10.1080/10428190500264496 16263578

[66] Jiménez M Martínez C Ercilla G Carreras E Urbano-Ispízua A Aymerich M Reduced-intensity conditioning regimen preserves thymic function in the early period after hematopoietic stem cell transplantation. Exp Hematol (2005) 33:1240–8. 10.1016/j.exphem.2005.06.016 16219547

[67] Busca A Lovisone E Aliberti S Locatelli F Serra A Scaravaglio P Immune reconstitution and early infectious complications following nonmyeloablative hematopoietic stem cell transplantation. Hematology (2003) 8:303–11. 10.1080/10245330310001612125 14530172

[68] Roll P Muhammad K Stuhler G Grigoleit U Einsele H Tony HP. Effect of ATG-F on B-cell reconstitution after hematopoietic stem cell transplantation. Eur J Haematol (2015) 95(6):514–23. 10.1111/ejh.12524 25677646

[69] Fuji S Kim SW Yano S Hagiwara S Nakamae H Hidaka M A prospective multicenter study of unrelated bone marrow transplants using a reduced-intensity conditioning regimen with low-dose ATG-F. Bone Marrow Transplant (2015) 51(3):451–3. 10.1038/bmt.2015.268 26551777

[70] Zand MS. B-cell activity of polyclonal antithymocyte globulins. Transplantation (2006) 82(11):1387–95. 10.1097/01.tp.0000244063.05338.27 17164703

[71] Booth C Lawson S Veys P. The current role of T cell depletion in paediatric stem cell transplantation. Br J Haematol (2013) 162:177–90. 10.1111/bjh.12400 23718232

