# Supplementary material for: Joint Modeling of Immune Reconstitution Post Haploidentical Stem Cell Transplantation in Pediatric Patients With Acute Leukemia Comparing CD34+-Selected to CD3/CD19-Depleted Grafts in a Retrospective Multicenter Study
Source: Front Immunol. 2018 Aug 14;9:1841. doi: 10.3389/fimmu.2018.01841 (PMC6102342; doi:10.3389/fimmu.2018.01841)
Supplement: Supplementary file 1 [file Data_Sheet_1.PDF]

Supplementary Material

Joint modeling of immune reconstitution post haploidentical stem cell transplantation in pediatric patients with acute leukemia comparing $CD34^+$-selected to CD3/CD19-depleted grafts in a multicenter trial

Emilia Salzmann-Manrique[1,2*], Melanie Bremm[2], Sabine Huenecke[2], Milena Stech[2], Andreas Orth[3], Matthias Eyrich[4], Ansgar Schulz[5], Ruth Esser[6], Thomas Klingebiel[2], Peter Bader[2], Eva Herrmann[1†], Ulrike Koehl[6,7†]

*** Correspondence:** Emilia.Salzmann@kgu.de

Suppl. Figure 1. Predicted cells immune reconstitution for ten years old children

The solid line with dark gray confidence region represents the immune recovery after haplo-PBSC$_{CD34sel}$ and the dashed lines with light gray confidence region the immune recovery after haplo- PBSC$_{CD3/CD19dep}$ for ten-year-old children.

Abbreviations: *ns*, not significant; *, *P*< 0.05; **, *P*< 0.01; ***, *P*< 0.001

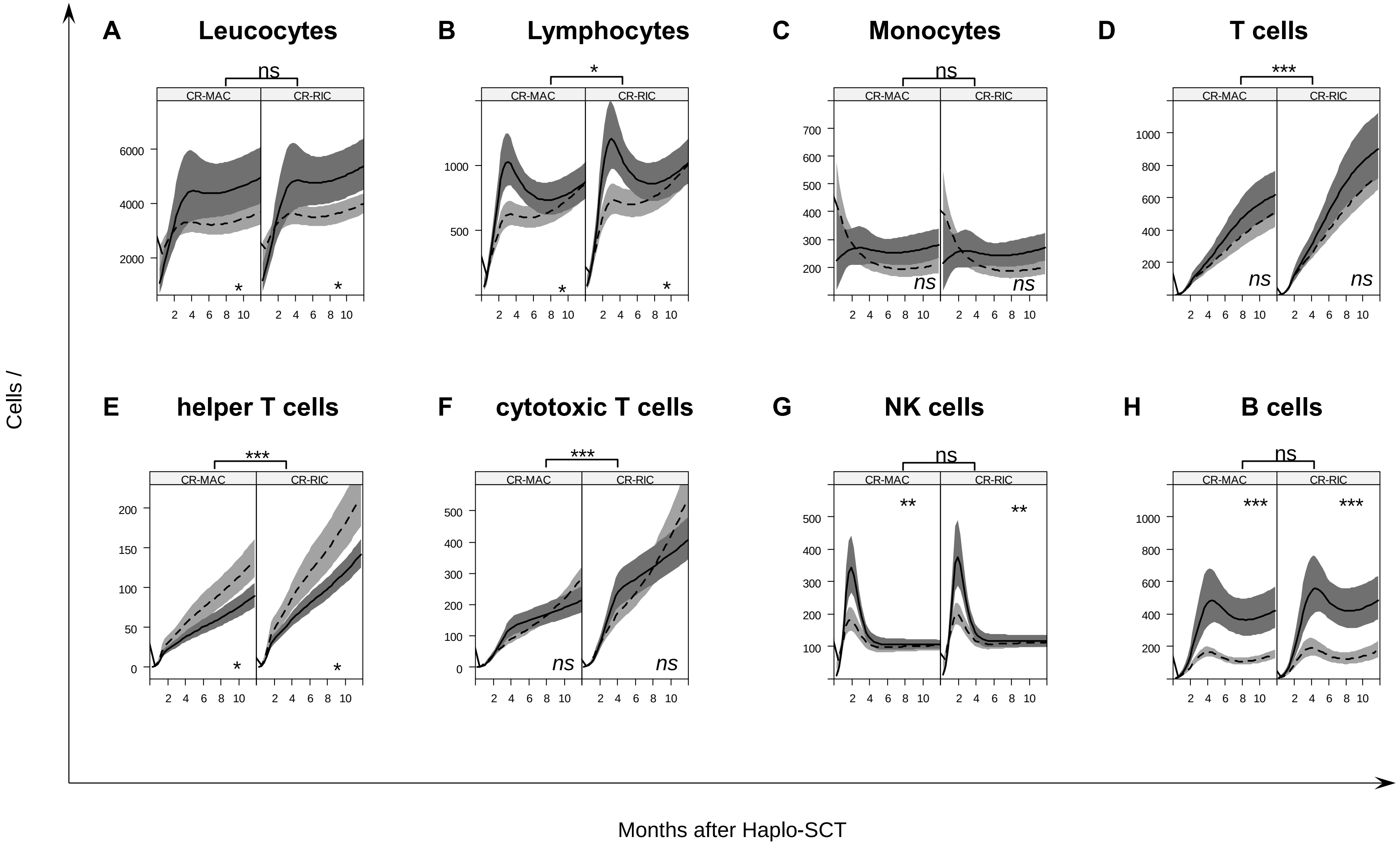

Suppl. Table 1 . Age-matched leukocytes subpopulation reference values in childhood published by Huenecke et al.

The table shows absolute cell count [cells/µl] of the predicted mean norm values for children and young adults for the considered leukocyte population [27].

Age (years)	*Leucocytes*	*Lymphocytes*	*Monocytes*	*$CD3^+$ T cells*	*$CD4^+$helper -T cells*	*$CD8^+$cytotoxic -T Cells*	*$CD56^+$ NK cells*	*$CD19^+$ B Cells*
0	9702	6685	1971	4581	3279	1210	256	1645
1	8965	5657	390	3832	2560	1079	252	1379
2	8385	4856	389	3265	2060	971	249	1165
3	7927	4231	389	2836	1713	881	245	992
4	7566	3743	389	2511	1472	806	242	852
5	7282	3363	389	2265	1305	745	238	740
6	7058	3067	389	2078	1189	694	234	648
7	6881	2835	389	1937	1108	652	232	575
8	6742	2655	389	1830	1052	617	228	516
9	6633	2514	389	1749	1013	588	225	468
10	6546	2405	389	1688	986	564	222	429
11	6478	2319	389	1641	967	544	219	398
12	6424	2252	389	1606	954	528	216	373
13	6382	2200	389	1579	945	514	213	353
14	6349	2160	389	1559	939	503	210	336
15	6322	2128	389	1544	934	494	207	323
16	6302	2103	389	1533	931	486	204	312
17	6285	2084	389	1524	929	480	201	304
18	6272	2069	389	1517	928	474	198	297
19	6262	2057	389	1512	926	470	195	291
20	6254	2048	389	1508	926	467	193	287

**Suppl. Table 2A.	Predicted cells immune reconstitution in absolute values [cells/µl] for ten years old children who received myeloablative conditioning regimen (MAC)**

The table shows the predicted absolute values arise from our joint model with 95% confidence interval at +30, +60, +90, +120 and +365 days after transplantation. The values were obtained for children in complete remission (CR) at time point of haplo-SCT.

Cells	***Day +30***		***Day +60***		***Day +90***		***Day +180***		***Day +365***	
	**$CD34^{+}$sel**	**CD3/CD19 dep**	**$CD34^{+}$sel**	**CD3/CD19 dep**	**$CD34^{+}$sel**	**CD3/CD19 dep**	**$CD34^{+}$sel**	**CD3/CD19 dep**	**$CD34^{+}$sel**	**CD3/CD19 dep**
Leucocytes	4862<br>(3949 – 5987)	3607<br>(3178 – 4093)	3372<br>(2494 – 4560)	3022<br>(2653 – 3443)	4128<br>(3026 – 5630)	3227<br>(2836 – 3671)	4291<br>(3410 – 5399)	3161<br>(2813 – 3553)	4862<br>(3949 – 5987)	3607<br>(3178 – 4093)
Lymphocytes	377<br>(298 – 478)	307<br>(260 – 362)	846<br>(689 – 1040)	527<br>(449 – 619)	1016<br>(841 – 1226)	619<br>(532 – 721)	762<br>(654 – 886)	602<br>(522 – 695)	876<br>(748 – 1026)	870<br>(754 – 1004)
Monocytes	252<br>(188 – 339)	336<br>(282 – 399)	267<br>(209- 340)	278<br>(235 – 329)	268<br>(209 – 344)	242<br>(205 – 286)	253<br>(211 – 302)	199<br>(171 – 230)	283<br>(235 – 341)	211<br>(180 – 246)
$CD3^{+}$	20<br>(14 – 28)	19<br>(15 – 24)	88<br>(63 – 123)	83<br>(66 – 104)	154<br>(118 – 202)	135<br>(110 – 164)	355<br>(284 – 445)	276<br>(228 – 335)	624<br>(504 – 774)	522<br>(427 – 638)
$CD4^{+}$	9<br>(7 – 11)	13<br>(9 – 18)	23<br>(19 – 28)	32<br>(24 – 41)	31<br>(26 – 37)	44<br>(36 – 54)	51<br>(42 – 61)	76<br>(61 – 94)	91<br>(77 – 109)	139<br>(116 – 165)
$CD8^{+}$	13<br>(10 – 16)	9<br>(6 – 13)	40<br>(35 – 46)	41<br>(31 – 54)	67<br>(59 – 76)	89<br>(72 – 111)	125<br>(110 – 141)	148<br>(120 – 184)	289<br>(257 – 326)	214<br>(177 – 259)
$CD3^{-}CD56^{+}$	197<br>(152 – 256)	145<br>(119 – 176)	323<br>(254 – 409)	175<br>(144 – 212)	189<br>(161 – 223)	131<br>(110 – 155)	106<br>(90 – 126)	98<br>(83 – 117)	106<br>(93 – 121)	103<br>(88 – 121)
$CD19^{+}$ B	12<br>(8 – 18)	7<br>(6 – 9)	45<br>(31 – 67)	22<br>(18 – 26)	92<br>(62 – 137)	37<br>(30 – 45)	105<br>(80 – 139)	31<br>(26 – 37)	109<br>(81 – 147)	40<br>(33 – 48)

Suppl. Table 2B. Predicted cells immune reconstitution in absolute values [cells/µl] for ten years old children who received reduce-intensity conditioning regimen (RIC)

The table shows the predicted absolute values arise from our joint model with 95% confidence interval at +30, +60, +90, +120 and +365 days after transplantation. The values were obtained for a 10 years-children in complete remission (CR) at time point of haplo-SCT.

Cells	***Day +30***		***Day +60***		***Day +90***		***Day +180***		***Day +365***	
	**$CD34^+$sel**	**CD3/CD19 dep**	**$CD34^+$sel**	**CD3/CD19 dep**	**$CD34^+$sel**	**CD3/CD19 dep**	**$CD34^+$sel**	**CD3/CD19 dep**	**$CD34^+$sel**	**CD3/CD19 dep**
Leucocytes	2438<br>(1896 – 3134)	2848<br>(2542 - 3190)	3739<br>(2866 – 4876)	3351<br>(2985 – 3761)	4577<br>(3488 – 6006)	3577<br>(3181 – 4023)	4758<br>(3940 – 5744)	3505<br>(3169 – 3876)	5391<br>(3643 – 6416)	3999<br>(3643 – 4389)
Lymphocytes	442<br>(345 – 567)	359<br>(305 – 424)	993<br>(791 – 1245)	618<br>(524 – 730)	1191<br>(966 – 1468)	726<br>(619 – 853)	893<br>(763 – 1046)	706<br>(605 – 924)	1027<br>(869 – 1214)	1020<br>(884 – 1177)
Monocytes	242<br>(181 – 323)	322<br>(275 – 377)	256<br>(200 – 327)	267<br>(227 – 313)	257<br>(200 – 331)	232<br>(197 – 273)	242<br>(205 – 287)	190<br>(165 – 219)	271<br>(226 – 326)	202<br>(176 – 231)
$CD3^+$	29<br>(20 – 41)	28<br>(24 – 34)	129<br>(93 – 180)	121<br>(101 – 145)	225<br>(173 – 294)	197<br>(169 – 229)	519<br>(418 – 645)	403<br>(350 – 465)	913<br>(733 – 1136)	763<br>(667 – 874)
$CD4^+$	14<br>(12 – 18)	21<br>(15 – 29)	36<br>(31 – 43)	51<br>(39 – 66)	49<br>(42 – 57)	70<br>(57 – 85)	81<br>(70 – 94)	121<br>(97 – 149)	146<br>(128 – 165)	221<br>(183 – 267)
$CD8^+$	24<br>(19 – 31)	16<br>(11 – 25)	77<br>(62 – 95)	78<br>(59 – 102)	128<br>(106 – 156)	171<br>(138 – 211)	238<br>(197 – 289)	283<br>(231 – 348)	553<br>(455 – 672)	409<br>(347 – 482)
$CD3^-CD56^+$	216<br>(165 – 283)	158<br>(135 – 185)	353<br>(276 – 452)	191<br>(163 – 225)	207<br>(175 – 245)	143<br>(123 – 165)	116<br>(98 – 138)	108<br>(93 – 125)	116<br>(100 – 135)	113<br>(101 – 127)
$CD19^+$ B	14<br>(9 – 21)	8<br>(6 – 11)	52<br>(36 – 75)	25<br>(19 – 34)	106<br>(73 – 153)	42<br>(32 – 57)	121<br>(94 – 155)	36<br>(27 – 47)	126<br>(96 – 165)	46<br>(35 – 61)

Suppl. Table 3. Log rank test was used to compare Kaplan-Meier estimates between different patients groups.

Compared Groups	Log. Rank Test
MAC-CD34sel vs. RIC-CD34sel	0.548
MAC-CD3/CD19dep vs. RIC-CD3/CD19dep	0.542
MAC-CD34sel vs. MAC-CD3/CD19dep	0.253
MAC-CD34sel vs. RIC-CD3/CD19dep	0.339
RIC-CD34sel vs. MAC-CD3/CD19dep	0.981
